# Internet of Things Smart Sunscreen Station: Descriptive Proof-of-Concept Study

**DOI:** 10.2196/17079

**Published:** 2020-05-28

**Authors:** Helen Ford, Jeremy Herbert, Caitlin Horsham, Alexander Wall, Elke Hacker

**Affiliations:** 1 Institute of Health and Biomedical Innovation School of Public Health and Social Work Queensland University of Technology Brisbane Australia; 2 Designworks Group Pty Ltd Brisbane Australia

**Keywords:** skin neoplasms, melanoma, health promotion, public health, preventive medicine, web applications

## Abstract

**Background:**

Skin cancer is the most prevalent but also most preventable cancer in Australia. Outdoor workers are at increased risk of developing skin cancer, and improvements in sun protection are needed. Sunscreen, when applied at the recommended concentration (2 mg/cm^2^), has been shown to block the harmful molecular effects of ultraviolet radiation in vivo. However, sunscreen is often not applied, reapplied sufficiently, or stored adequately to yield protection and reduce sunburns.

**Objective:**

The primary aim of this study was to test an Internet of Things approach by deploying a smart sunscreen station to an outdoor regional mining site.

**Methods:**

We deployed a smart sunscreen station and examined the key technological considerations including connectivity, security, and data management systems.

**Results:**

The smart sunscreen station was deployed for 12 days at a mining workplace (Dalby, Australia). The smart sunscreen station’s electrical components remained operational during field testing, and data were received by the message queuing telemetry transport server automatically at the end of each day of field testing (12/12 days, 100% connectivity).

**Conclusions:**

This study highlights that an Internet of Things technology approach can successfully measure sunscreen usage and temperature storage conditions.

## Introduction

Ultraviolet radiation (UVR) is the main environmental risk factor for melanoma and keratinocyte skin cancers. Australia has one of the highest rates of skin cancers in the world, which are at least double those of the United States or United Kingdom [1]. A meta-analysis has shown that outdoor workers are at increased risk of developing skin cancer, with the risk of squamous-cell carcinoma nearly double for outdoor workers than for indoor workers [2], while the risk of basal cell carcinoma is increased by almost 1.5 times [3]. In the Australian population, approximately 23.1% of all workers are estimated to be exposed to UVR at work [4]. Despite continuing educational efforts, a disconnect persists between public understanding of the harmful effects of excessive sun exposure and regular use of sunscreen. The Australian Work Exposures Study reported on 1100 outdoor workers and found that, although sun protection was used by 94.9% of Australian outdoor workers, only 8.7% of workers were classified as fully protected, with the most frequently used methods being protective clothing and hats [4]. In an Australian outdoor workers study (n=162), 93% of workers reported sunscreen was provided by their workplace; however, sunscreen was the least frequently used personal protective equipment (PPE), with only 40% of workers self-reporting usually or always using sunscreen during work hours [5]. There was also a high percentage of outdoor workers (56.9%) exposed to UVR at work for more than 4 hours per day, with occupations such as mining, farming, and animal and horticultural workers [4]. Skin cancer has been listed as a work-related condition requiring priority for prevention activities in the Australian Work Health and Safety Strategy 2012-2022, and PPE is required to be provided by the workplace [6].

Regular sunscreen application has been shown to reduce the incidence of squamous cell carcinomas and melanoma [7] and block the harmful molecular effects of UVR on skin cells in vivo [8]. Health interventions to improve sun protection behaviors often assess sunscreen usage through self-report, which can be subject to social desirability bias and inaccurate memory recall [9,10]. Questionnaires or interviews can also be burdensome to complete, participants may miss questions, and they do not allow for real-time monitoring of compliance. Objective measurements of sunscreen usage are possible by using standardized scales and weighing bottles to determine the amount used. Limitations of the weighing method include the lack of a time stamp on usage data, reliance on the return of the sunscreen bottle for weighing, and inability to account for how many people may be using the sunscreen. Armstrong and colleagues [11] previously developed an electronic monitoring device to measure sunscreen usage, which involved a mobile phone being strapped to a sunscreen bottle. Each time the cap on the sunscreen tube was removed, the electronic monitor sent an SMS message in real time to a database where the dates and times of openings were recorded. However, the device was bulky to carry, and the database interface was limited [11].

The Internet of Things (IoT) is a concept where ordinary items are upgraded to include internet connectivity, allowing them to transmit information. In the health system, IoT-enabled smart devices have been used for preventative health in a US hospital to measure how often hospital workers were washing their hands [12]. The system had a ceiling-mounted sensor to monitor the flow of people as well as soap and hand sanitizer electronic monitoring dispensers to track usage. This provided hospital managers with a compliance rate of how many people washed their hands upon entering certain areas.

To improve sunscreen application in the workplace, this study aimed to leverage IoT-enabled smart devices by developing a smart sunscreen station that can stream data to an online management system to assist health and safety managers in determining the frequency of sunscreen use, measuring the temperature where the sunscreen is kept, and being alerted when sunscreen containers need replacing.

## Methods

### Smart Sunscreen Station Development

**Figure 1:**
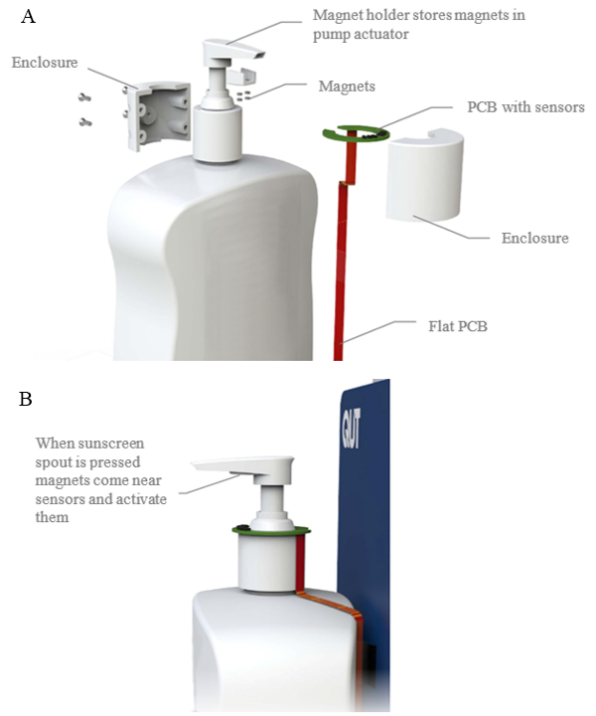
Smart sunscreen station measuring sunscreen dispensing with (A) retrofitted sunscreen sensor assembly and (B) pump mechanism and sensor detection concept. PCB: printed circuit board.

**Figure 2:**
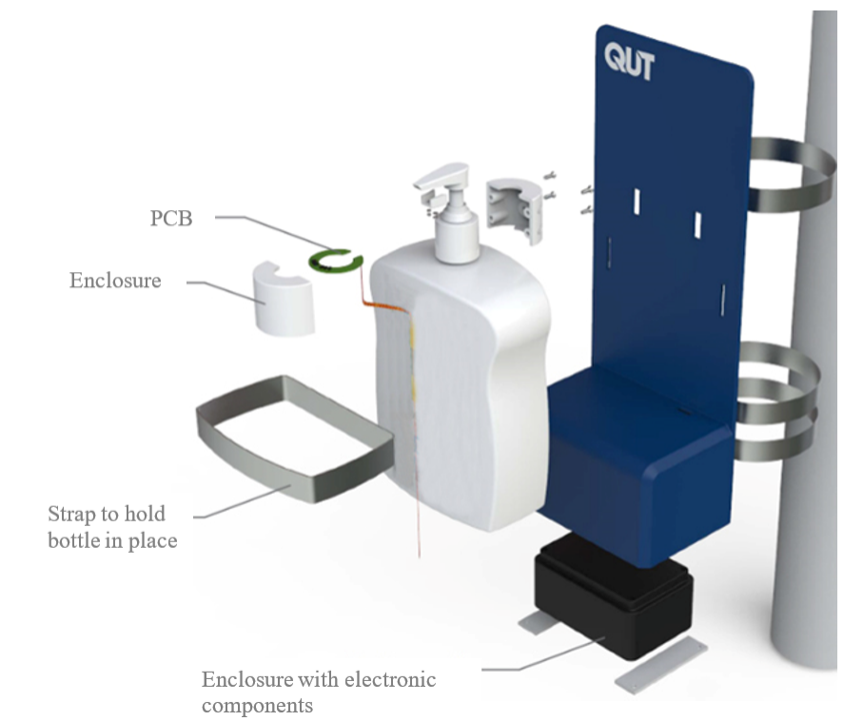
Smart sunscreen station housing with electrical components housed within the bottom of the stand and stainless steel strapping to secure the bottle in place. PCB: printed circuit board.

**Figure 3:**
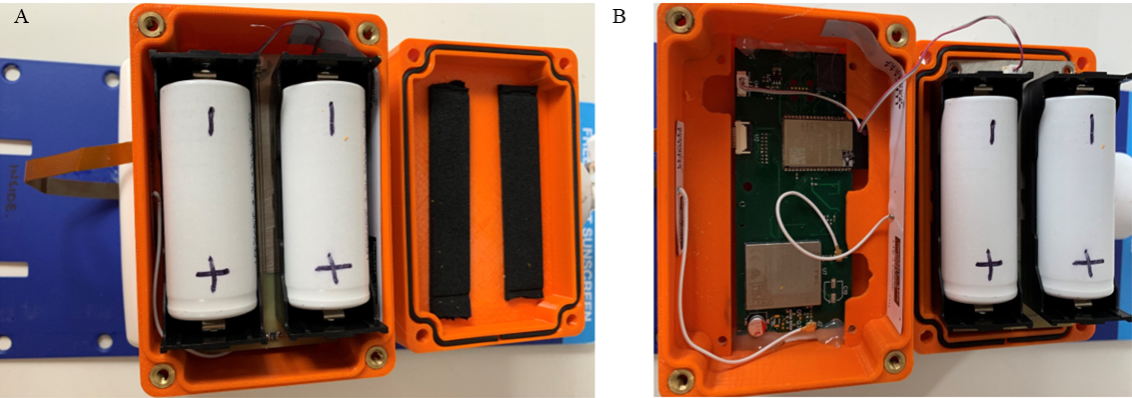
Smart sunscreen station electronic components; the battery housing was protected from water and dust using rubber seals (A), and the BG96 module was connected to a narrowband Internet of Things subscriber identification module (SIM) card and located underneath the battery platform with one NB1 radio antenna attached to the wall of the housing (B).

**Figure 4:**
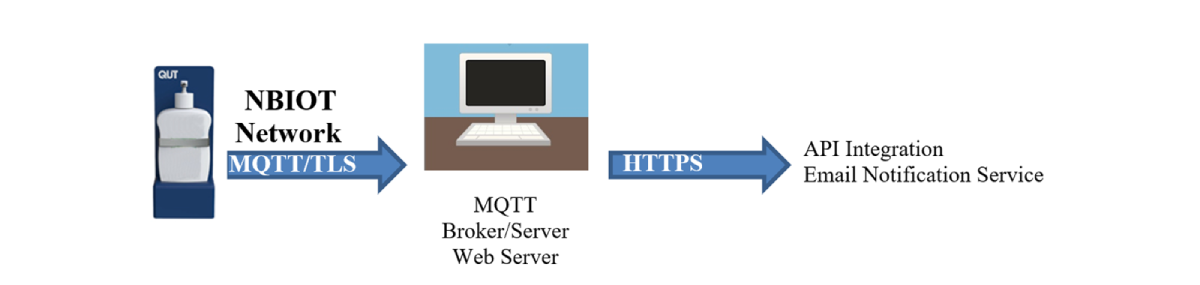
The process flow of data from an Internet of Things (IoT) smart sunscreen station. API: application programming interface; MQTT: message queuing telemetry transport; NBIOT: narrowband Internet of Things; TLS: Transaction Layer Security.

This study developed an IoT smart sunscreen station that objectively measures the use of a commercially available 1-L pump sunscreen product. The commercially available sunscreen product was retrofitted with magnets and flexible printed circuit board sensors to measure the pushing of the pump mechanism and dispensing of sunscreen (Figure 1). The sunscreen sensor assembly was then connected via a flat flex cable to a printed circuit board inside the smart sunscreen station housing that contained batteries, a main microcontroller (ESP32, Espressif, Shanghai, China), a cellular Cat-NB1 radio (BG96, Quectel Wireless Solutions Co Ltd, Shanghai, China), and processing electronics. The housing was constructed from plastic to allow the cellular antennas to be mounted internally to protect them from exposure to the environment (Figure 2). Cellular connectivity was provided via a Cat-NB1 network (Telstra Corporation Limited, Melbourne, Australia). An open-source message queuing telemetry transport broker/server (Mosquitto, Eclipse Foundation, Ottawa, Canada) was used to collect the data from the device over the internet (Figure 3), and all communication between the device and server was encrypted using Transaction Layer Security (TLS) version 1.2 (Figure 4). Data were stored in a SQL database and served using a small web application written in Python v3.

### Observational Laboratory Testing

To check the smart sunscreen station was connecting and recording data to the server correctly, observational testing was performed in Brisbane, Australia (approximate latitude 27 ^o^S, 153 ^o^E). A full 1-L sunscreen bottle was pumped 10 times, and the bottle was weighed to confirm the volume of sunscreen dispensed. A half-empty 1-L sunscreen bottle with a net weight of 500 g was pumped 10 times and weighed. A nearly empty 1-L sunscreen bottle with a net weight of 200 g was pumped 10 times and weighed. The time-stamped data on the server were then compared with the observed time-recorded pumps and weight of the bottle.

The smart sunscreen station underwent heat testing by placing the unit in an oven at 35 ^o^C for 1 hour. The temperature of the smart sunscreen station was recorded using an infrared handheld thermometer (ThermaTwin TN410LCE Infrared Thermometer, OneTemp, Adelaide, Australia). The smart sunscreen station was further heat tested by placing it in an oven at 45 ^o^C for 30 minutes, and data were recorded for an additional 12 days.

### Smart Sunscreen Station Deployment

The field study was conducted during November 2018 to December 2018 (summer in Australia) when the UV Index in Queensland is consistently above 9 and can reach 14+, requiring sun protection every day. One smart sunscreen station was deployed to an outdoor workplace located in Dalby, Australia (latitude 27 ^o^S, 151 ^o^E). The smart sunscreen station was taken out to drilling rig sites located between Dalby and Chinchilla during the testing period of November 25, 2018 to December 6, 2018. The site was an operational gas mining venture that had worker activity in a high UV environment and no underground workforce. The smart sunscreen station was placed in the air-conditioned meeting and lunch room, which is a high traffic area that is accessed by all workers at the start of the day and during breaks. This is where existing safety talks are held before commencing outdoor work. The sunscreen used in this study was commercially available SPF 50+ (the highest SPF level available in Australia). The smart sunscreen station did not monitor personal use of sunscreen or who had used it. It measured the number of times the sunscreen was pumped and the storage temperature, and it sent notifications to the database when it required sunscreen replacement.

Feedback was obtained from end users including health and safety officers, purchasing officers, and health officers to design and refine the software dashboard. Questions surrounding the dashboard infographics were asked to assist with communicating the data collected on the IoT smart sunscreen station. In addition, email and phone contact details of the researchers were provided to the workers for complaints, technical issues, or further information during the deployment. The deployment of the smart sunscreen station was to assess the functionality and not human subjects’ research; therefore, we obtained institutional ethics review board exemption.

### Statistical Analysis

Sunscreen usage variables were dichotomized to categorical data: Sunscreen usage data were coded “yes” if the timestamped data of sunscreen use were recorded to the server and coded “no” if the sunscreen bottle was pumped but data were not recorded to the server. The Cohen kappa score was calculated to determine if there was agreement between categorical variables for sunscreen usage. Sunscreen weight data were recorded in grams, and the observed weight per pump was calculated and compared to the server-predicted weight. Spearman’s rank correlation coefficient was used to determine correlations between server-predicted and observed weight. Values >0.4-0.6 were considered moderate, >0.6-0.8 substantial, and >0.8-1.0 almost perfect agreement. Prism graph-pad (version 7, GraphPad Software, San Diego, CA) and SPSS software (version 25.0, IBM Corp, Armonk, NY) were used for analyses.

## Results

### Laboratory Testing and Connectivity

Laboratory testing of the smart sunscreen station demonstrated there was perfect agreement between observed sunscreen use and server-recorded sunscreen use (κ=1.0, 95% CI 1.00-1.00; Table 1). There was almost perfect agreement between the observed weight of the sunscreen bottle and predicted weight based off server usage data (r=1.0, 95% CI 1.00-1.00, *P*<.001; Table 1). The smart sunscreen station temperature sensor recorded to the server that the temperature exceeded the maximum storage temperature of 30 ^o^C following a 1-hour oven incubation at 35 ^o^C. The unit was tested further with a 1-hour oven incubation at 45 ^o^C, and the unit continued to be operational and connect to the server and send data during the post-heat stress measurement period (100% connectivity, 12/12 days).

**Table 1:** Agreement between observed sunscreen usage and server-recorded measurements.

Sunscreen bottle status	Sunscreen pump recorded in the server		Observed average weight per pump (g)	Server-predicted weight per pump (g)
	Yes, n (%)	No, n (%)		
Full bottle weighing 1000 g bottle (n=10 pumps)	10 (100)	0 (0%)	1.732	1.60
Half-empty bottle weighing 500 g (n=10 pumps)	10 (100)	0 (0%)	1.564	1.55
Nearly empty bottle weighing 200 g (n=10 pumps)	10 (100)	0 (0%)	1.526	1.50

### Field Testing and Connectivity

**Figure 5:**
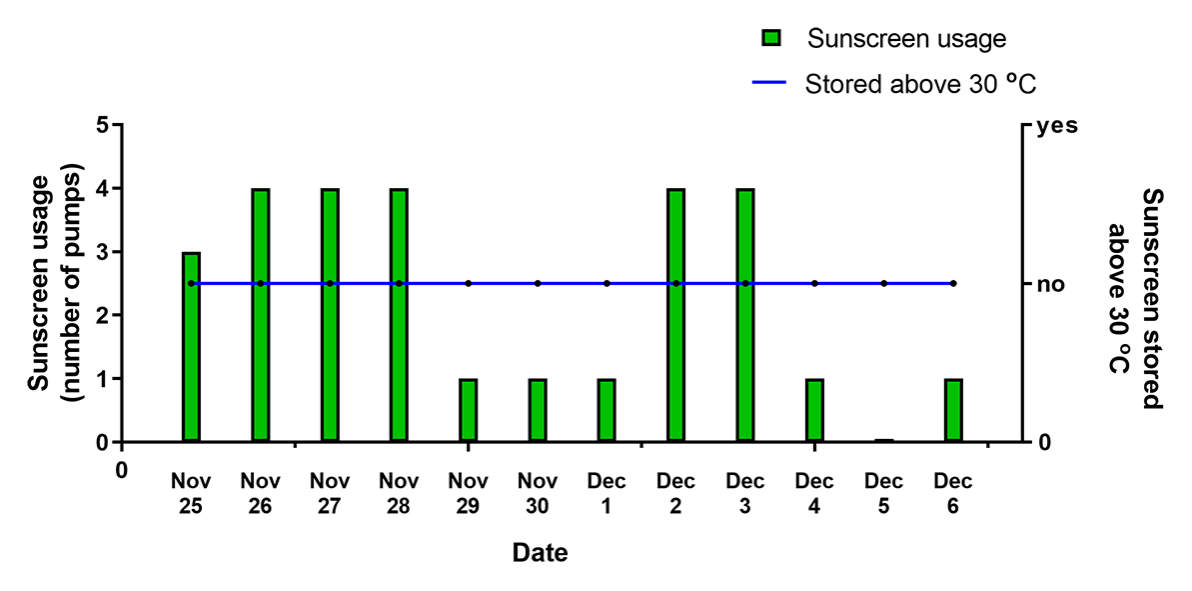
Data received each day from the smart sunscreen station, including number of pumps and temperature status.

**Figure 6:**
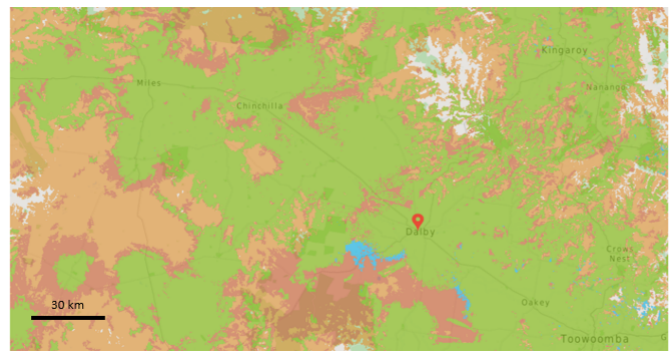
Network coverage for the field testing site in Dalby, Queensland (green, good 4G reception; blue, slower 4G reception; red, 3G reception only; orange, external antenna required for reception). Image adapted from [13].

The smart sunscreen station uploaded data each day to the message queuing telemetry transport server during the outdoor workplace deployment (Figure 5). No complaints nor concerns were logged from the workforce during the 12 days the smart sunscreen station was deployed. The smart sunscreen station was kept in an air-conditioned site meeting and lunch room at a drill rig site and did not exceed the maximum storage temperature of 30 ^o^C during the field test. The network coverage for the region where the smart sunscreen station was located illustrated good reception as shown by the green areas in the map (Figure 6). The smart sunscreen station recorded data locally each day, and data were uploaded to the server once each evening as programmed by the software, with data received for 12 of the 12 days (100% connectivity). The smart sunscreen station did not require continuous connectivity and stored data locally, only transmitting data once a day. The data transmission took approximately 10 seconds, with a 70-mA average transmit current as measured on the device. Sleep current was measured at approximately 10-20 uA. As a result, in normal conditions, the system battery life is expected to be predominantly limited by self-discharge of the lithium iron phosphate (LiFePO_4_) batteries.

### Security

All communications over the internet were protected using TLS v1.2 with a preshared certificate in the firmware of the device. Encryption was performed on the microcontroller itself to prevent the data being read or modified using a man-in-the-middle attack between the microcontroller and the cellular radio. In addition, the microcontroller utilized flash encryption and secure boot to prevent any unauthorized readout or modification of the firmware.

### Data Management Systems

**Figure 7:**
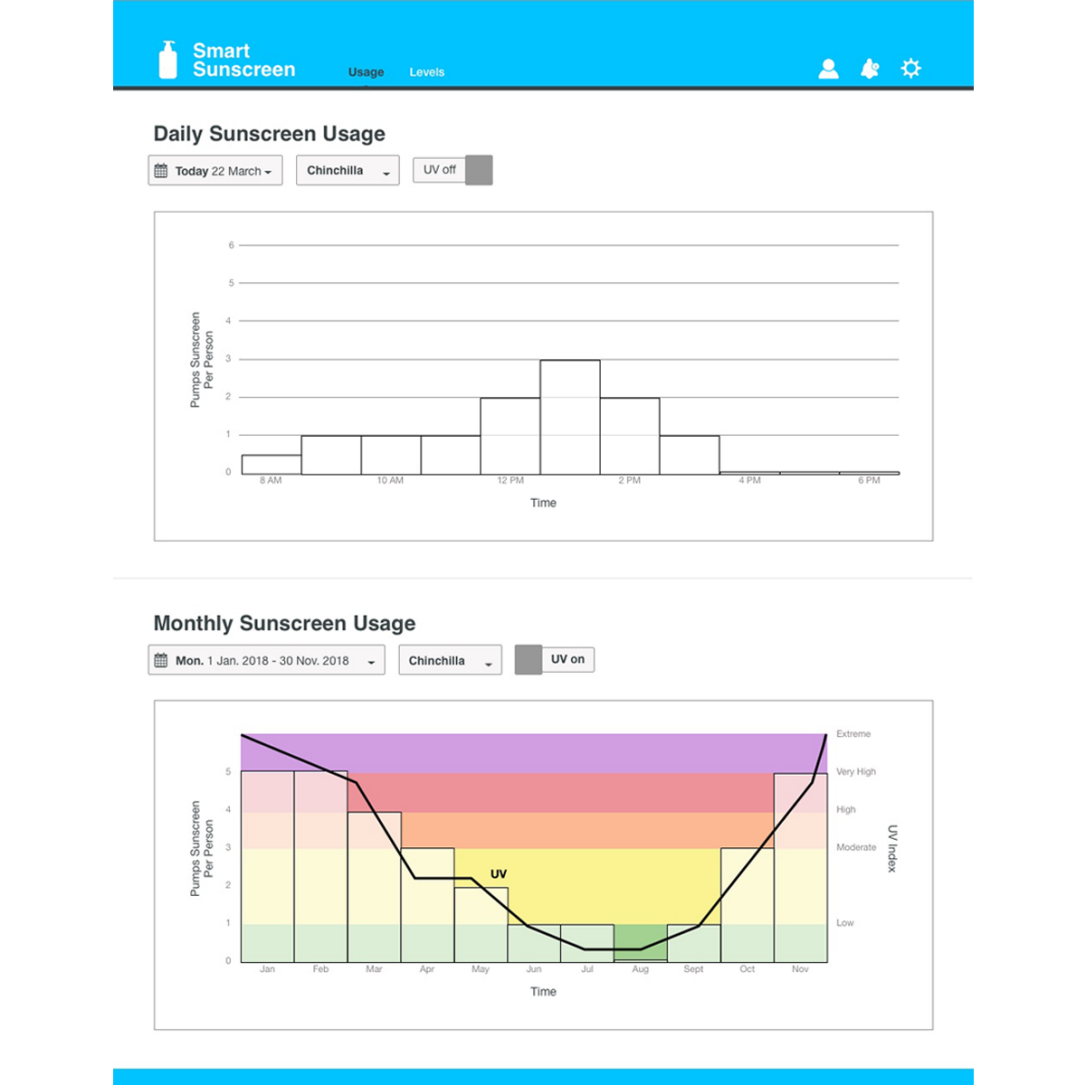
Dashboard infographics communicating smart sunscreen station data, including the daily sunscreen usage per person and the monthly sunscreen usage overlaid with UV index weather data.

**Figure 8:**
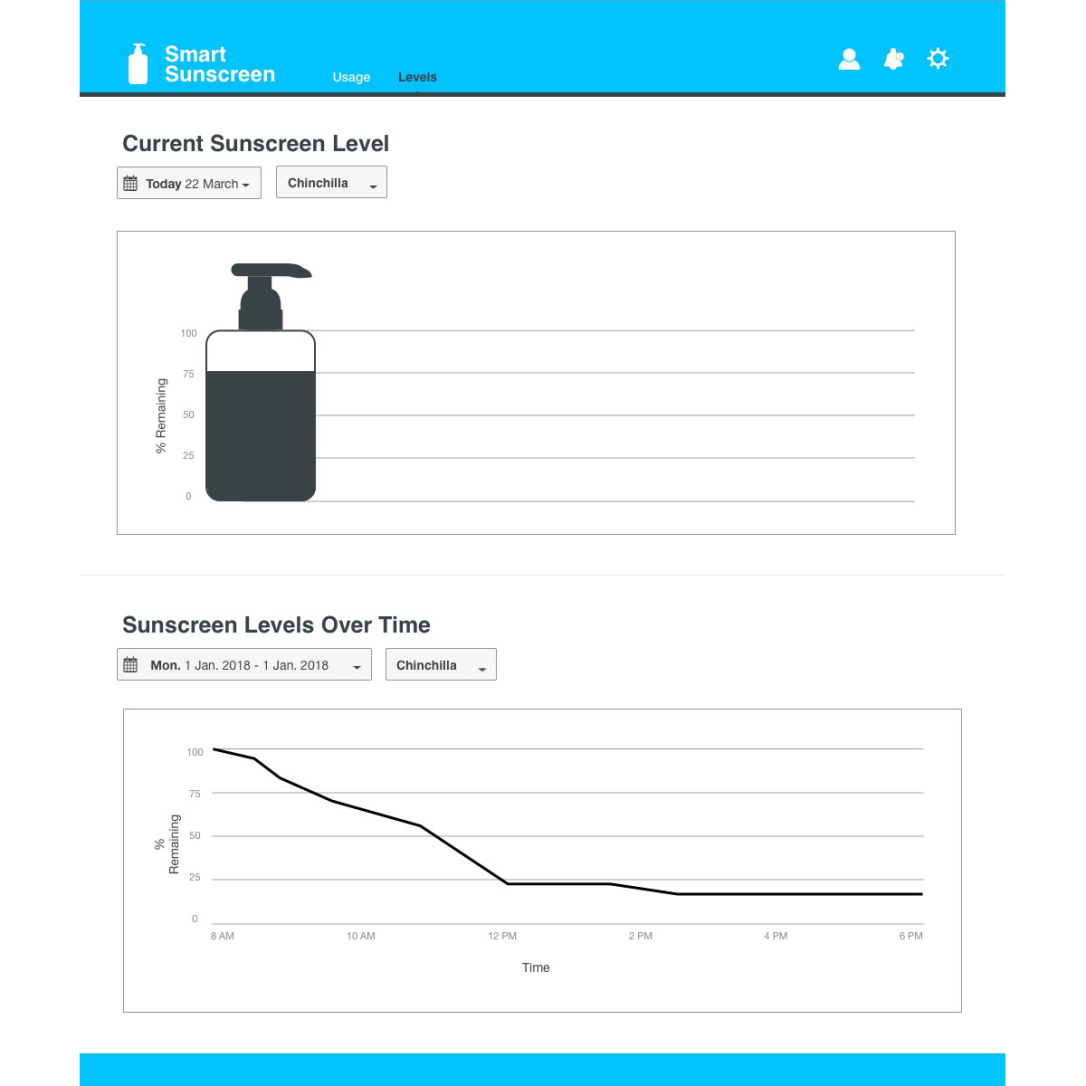
Smart sunscreen station dashboard data showing the current sunscreen bottle level the level of sunscreen in the bottle over time.

**Figure 9:**
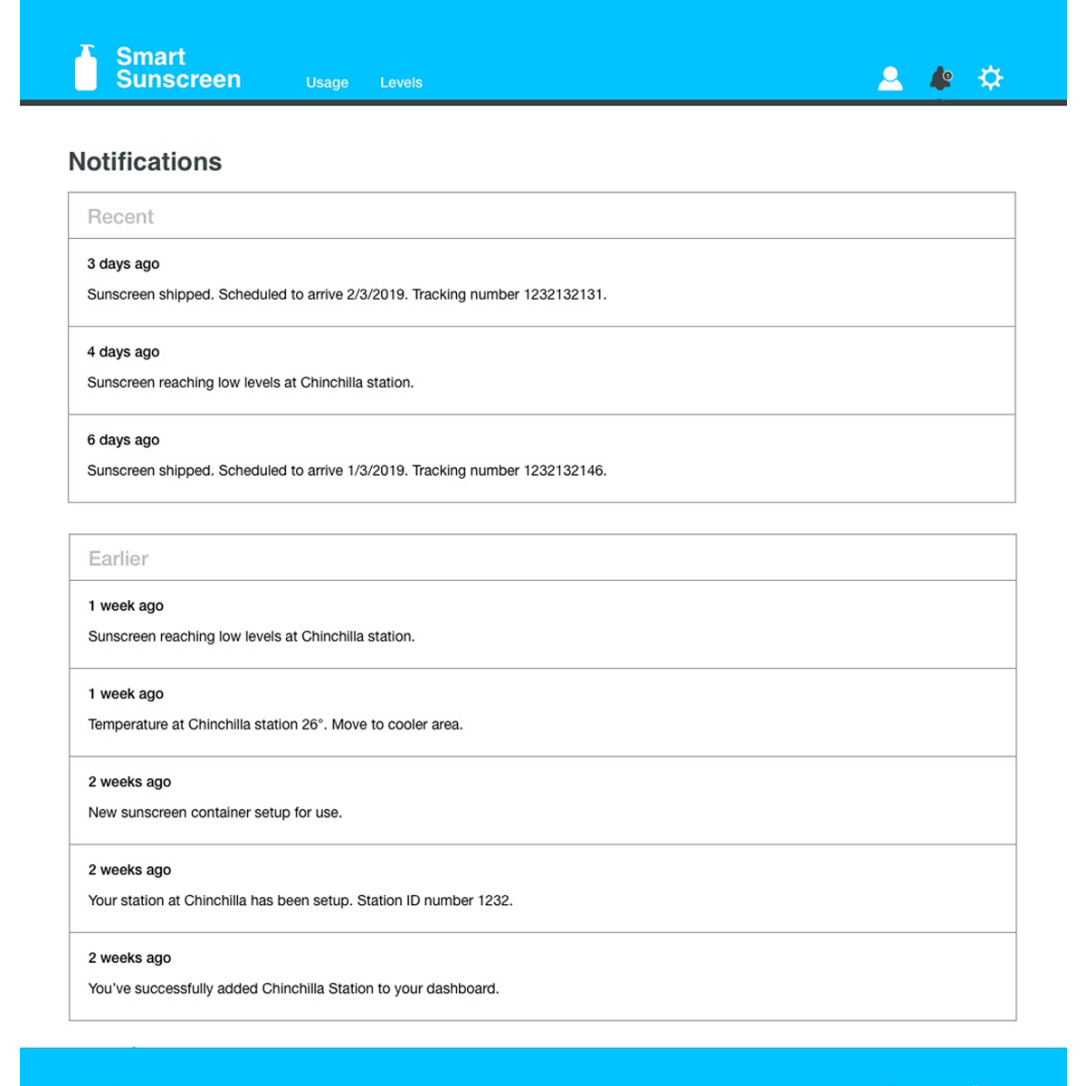
Smart sunscreen station dashboard showing notifications and alerts.

In consultation with stakeholders from health and safety roles at outdoor workplaces (n=5), dashboard infographics were developed to assist with communicating the data collected on the smart sunscreen station (Figures 7-9). Sunscreen usage data were converted into consumer-friendly infographics with the number of sunscreen pumps plotted over time using the time-stamp information collected. Historical data for previous months can be displayed and overlaid with color-coded UV Index weather information to illustrate trends in sunscreen usage (Figure 7). The level of sunscreen remaining in each bottle can be estimated using the number of pumps multiplied by the weight dispensed, which is calculated by the data management system and converted into an infographic (Figure 8). The level of sunscreen can then be plotted over time to assist with maintenance scheduling, and notifications are sent to alert users when sunscreen bottles need to be changed (Figures 8 and 9).

## Discussion

### Principal Findings

Our data demonstrated that this IoT smart sunscreen station can accurately record time-stamped sunscreen use from a 1-L bottle in an outdoor worker setting. During the 12-day field testing deployment in an outdoor workplace in regional Australia, the station’s electrical components remained operational, and data were received at the server each day, resulting in 100% connectivity. In addition, we reported a high correlation between observed sunscreen use and data recorded in the server as well as the observed weight of the sunscreen bottle and the predicted weight generated by the software based from the server usage data. Based on the study settings and results, we suggest that the smart sunscreen station may become a valuable tool to help optimize sunscreen supply and maintenance in an outdoor workplace.

Outdoor workers are at risk of developing skin cancer and are typically not using adequate sun protection, with studies showing that workers who perceived sun protection as valued in their workplace had higher levels of sun protection [14,15]. Uptake of sun protection by outdoor workers is affected by both workplace and personal factors, and strategies should target the workplace environment and workers' attitudes and behaviors [5].The provision of PPE by the workplace has been suggested to improve sun protection in outdoor workers by providing broader support to promote positive behaviors [16]. The smart sunscreen station shows significant promise to generate relevant data for workplace health and safety legislative requirements by illustrating that appropriate PPE is being supplied to workers. The real-time monitoring and inventory management of sunscreen would ensure appropriate levels of sunscreen are always available to workers, allow targeted training as part of health and safety programs in low usage areas, and ensure the sunscreen provided has not been overheated leading to reduced effectiveness.

Previous research has found low usage of sunscreen stations in public settings [17]. Kirby et al [17] implemented a free sunscreen station (which did not have IoT technology) at an amusement park, and the researchers observed usage from a distance by counting the number of people who passed, approached, or interacted with the sunscreen station. During the 3 observation periods, 879 people passed the sunscreen station, 2.6%-5.1% of visitors showed interest in the station, and even fewer visitors (0%-2.9%) used the sunscreen station. This study by Kirby et al [17] reported that members of the public had trouble recognizing the sunscreen station and suggested increased signage. In the outdoor workplace setting, sunscreen forms an important PPE measure, and data on sunscreen usage could be used to inform workforce PPE compliance and practices. A British study of outdoor workers reported that sun protection education was associated with workers' use of sun protective measures [18]. Other barriers to sunscreen use include that it is time-consuming and wearing it may be uncomfortable due to greasiness or increased sweatiness. Testing a variety of sunscreens using the smart sunscreen station to determine a preferred type could increase sunscreen use.

The IoT technology would also enable objective data collection rather than relying on self-report or conducting observational research in settings evaluating health promotion programs targeted to increase sun protection habits. Future studies will enable not only outdoor workplaces but also other high UV environments such as recreational venues and schools to evaluate sunscreen usage.

Ensuring secure and reliable network connectivity is an important consideration in IoT deployments, particularly in rural or regional areas. Unlike streaming applications, the IoT smart sunscreen station does not require continuous connectivity, as the device only needs to transmit data once per day; therefore, battery life can be conserved, and the servicing requirements of the IoT device are minimal. However, the data transmission is versatile and can be adjusted to range from seconds to days. For example, in public spaces or workplaces with a high volume of passing traffic, 5-minute data transmission intervals could be programmed, and this would allow replacement of sunscreen in a timely manner.

The narrowband IoT network was able to provide wide coverage when compared to other network alternatives. During this study, we demonstrated reliable connectivity in diverse areas from city centers to regional districts. The advancements in publicly available cloud-based services such as Amazon Web Services and Microsoft Azure for IoT devices also provide a cost-competitive alternative to traditional, on-premise hosting environments. These platforms allow users to connect their assets using industry standard security systems (such as TLS) to safely and securely gather data and undertake bidirectional communication.

The backend data management system processed the data received from the IoT device and was an important part of the design and crucial in allowing the data to be interpreted by the end user. This study found the use of infographics to convey IoT data to end users was acceptable and preferred over tables. These results are similar to other reports in health care communication, which show that engaging methods of visually communicating information in a colorful and concise manner are preferred [19]. The smart sunscreen device may utilize a lease arrangement with the options of 12-month or 24-month subscriptions, which include servicing and maintenance of the units and monthly scheduling of fees. This reduces the upfront prohibitive cost for the workplace to purchase the smart sunscreen station outright.

IoT has the potential to collect and integrate data in real time to improve operational efficiencies. However, to our knowledge, no previous studies have investigated the effective integration and application of IoT for sunscreen monitoring in an outdoor workplace. Previous work by Wu and colleagues [20] using an IoT wearable body sensor measured body temperature, heart rate, and environmental conditions such as temperature, carbon dioxide, UV Index, and humidity, and the data collected were used to trigger an alert if any emergency situation occurred. The body sensor data were connected using a low-power wide-area network, and data transmission was restricted to occur only within the gateway network area, which was reported to be 520 m indoors and 926 m when outdoors. The study was based on construction workers, reported high UVR exposure, and demonstrated the potential to gather safety monitoring information to assist workplace health and safety programs [20]. IoT technology has also previously been deployed as a notification system to measure restroom cleanliness in hospitals, whereby public users press the button when the restroom requires cleaning [21]. Chai and colleagues [21] reported this was a feasible method to streamline maintenance activities like restroom cleaning and would be further extended to include critical supply restocking tasks. Unlocking the potential for IoT technologies to assist and lead to evidence-based improvements requires evaluation of the impact of devices as well as strategies to engage end users and communicate the benefits yielded from the data collected. Key technical considerations for a successful deployment include ensuring appropriate network connectivity, designing a product with a robust management system that operates with current organizational platforms, planning for storage in high UV environments, and carefully considering the configuration to minimize privacy and security risks.

The aim of this study was to assess the proof of concept of a smart sunscreen station in a regional outdoor workplace. Limitations of the study include that we did not analyze the software data nor capture data on health behavior change such as the sun protection habit index or employee engagement; in addition, the duration of field testing was only 12 days. The interview analysis was limited by a small sample size (n=5), and future research could expand on additional enablers and barriers to sunscreen use by outdoor workers. Future projects deploying IoT devices could also explore further implementation factors including human aspects such as attitudes, behavior, and capacity as well as organizational, financial, and legislative processes.

### Conclusion

Consistent use of sunscreen is recommended to prevent sunburn and reduce the risk of developing skin cancers. Sunscreen is an essential PPE for outdoor workers, particularly on the face, neck, and hands. To assist workplaces to protect their workers from UVR, the smart sunscreen station could help streamline supply, restocking, and storage requirements, especially in high-temperature environments, as well as benefit health and safety programs with targeted training. This study provides evidence for the technical feasibility of IoT smart sunscreen stations in outdoor workplaces including regional and remote areas of Australia.

## Abbreviations

API: application programming interface.
IoT: Internet of Things.
MQTT: message queuing telemetry transport.
NBIoT: narrowband Internet of Things.
PCB: printed circuit board.
PPE: personal protective equipment.
TLS: Transaction Layer Security.
UVR: ultraviolet radiation.
